# New Hospital for Women, Euston Road

**Published:** 1905-11-11

**Authors:** 


### NEW HOSPITAL FOR WOMEN, EUSTON ROAD.

On Friday, November 3, a large company of the friends and subscribers of the hospital assembled to witness the opening of a new ward. The chair was taken by Mr. A. Gordon Pollock, member of the Managing Committee. The Rev. Llewellyn Davies, formerly chaplain to the hospital, gave a short sketch of the history of the institution, which was, he said, a monument of the energy and devotion of Mrs. Garrett Anderson. About forty years ago she opened a dispensary for women in Marylebone Road, and five years later its place was taken by a hospital containing only ten beds. In 1889 the foundation stone of the present hospital was laid by Queen Alexandra, then Princess of Wales. A cancer ward, nurses' home, and an operating theatre had been added from time to time, and now the present extension was found necessary.

Miss Webb, M.B., as head of the out-patient department, said that they had realised for a long time that a large proportion of the out-patients could not be cured without a short period of rest or surgical treatment, and therefore, it had been decided to open another new ward for the accommodation of these patients.

The Archbishop of Canterbury said that no one could possibly doubt that this hospital, which had at first been in the nature of an experiment, had proved to be a great gain to the forces engaged in the relief of suffering. It had at one time been an experiment, because it was conducted by women for women, but it was now welcomed into the fellowship of all the great hospitals. That hospitals should be more brought before the minds of the healthy was one of the crying needs of the day. All who cared at all for the alleviation of suffering should feel grateful to the pioneers who founded this hospital, where women were cared for by women with all the skill and tenderness which womanhood could command. Speaking as one who had had an unusual experience of illness, having been laid up seven or eight times for very considerable periods, his Grace said he had learnt to know the value of good nursing and loving care in illness. Hospitals like this enabled people, who otherwise would never have enjoyed such care, to carry away pleasant memories of the kindness they had experienced whilst they were ill. Patients often felt, when recovered, that after all they had had a good time and had seen a side of life different to that which came uppermost in the rough and tumble of a healthy existence.

The Chairman announced that the Archbishop had consented to become a patron of the hospital.

The new ward, which contains seven beds and a cot, is situated at the top of the building and is of circular shape.

